# Glances at the Hospitals

**Published:** 1904-08-06

**Authors:** 


### GLANCES AT THE HOSPITALS.

### SUSSEX COUNTY HOSPITAL, BRIGHTON.

The ordinary receipts from all sources, exclusive of legacies, were £9,069, and the ordinary expenditure was £15,815, which shows that a deficit of £6,746 existed. This is sufficiently alarming, and it justifies the Governors' statement that the hospital is in another financial crisis, and that prompt action is needed. In fact, it is clear to everyone that unless timely help is forthcoming, the only solution of the difficulty will be to close some of the wards, and this is always a painful step to contemplate; but it is imperative in the case of the Brighton Hospital. On looking over the cash account, we notice that the workmen's boxes produced only £544—a sum which is extremely small for a rich and populous county like Sussex; but even if the income from this source could be increased to a reasonable amount the total would still fall far short of the sum required. A nursing institute is attached to the hospital, and there are 29 nurses on its staff. After paying bonuses to secretary, matron, and nurses, a sum of £405 was carried to the general account. At the beginning of the year 185 patients were in residence, and 2,017 were admitted during it, making a total of 2,202 under treatment. The daily average number resident was 173, and the average stay in the hospital was 27½ days. We did not find any statement relative to the cost per head, or the cost per bed occupied. Of the total number of in-patients 990 were discharged cured, 749 relieved, 111 were discharged for other reasons, 169 died, and 184 remained under treatment. The medical and surgical tables merely show the numbers treated for various diseases, and do not give results. For instance, it is stated that 53 operations were performed for the radical cure of hernia, and 13 for strangulated hernia, but nothing is said of the numbers cured, relieved or died. No anæsthetic table is given.

